# Supplementary material for: Fully Human Monoclonal Antibodies Effectively Neutralizing Botulinum Neurotoxin Serotype B
Source: Toxins (Basel). 2020 May 7;12(5):302. doi: 10.3390/toxins12050302 (PMC7291131; doi:10.3390/toxins12050302)
Supplement: Supplementary file 1 [file toxins-12-00302-s001.pdf]

Supplementary Materials: Fully Human Monoclonal Antibodies Effectively Neutralizing Botulinum Neurotoxin Serotype B

Takuhiro Matsumura, Sho Amatsu, Ryo Misaki, Masahiro Yutani, Anariwa Du, Tomoko Kohda, Kazuhito Fujiyama, Kazuyoshi Ikuta and Yukako Fujinaga

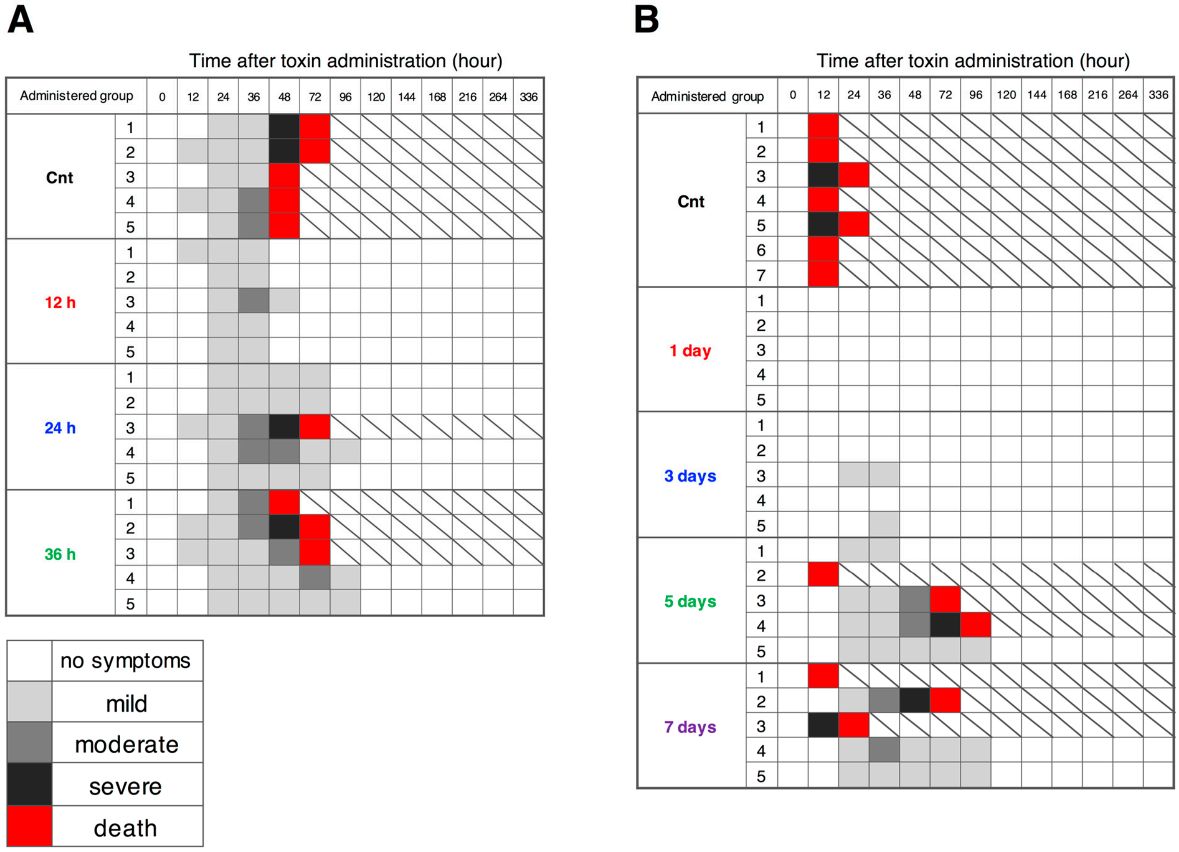

Figure S1. Therapeutic and preventive effects of HuMAbs (M2+M4) against BoNT/B1 intoxication: Changes of botulism symptoms in each mouse in the period of observation (2 weeks). (**A**) Post-exposure treatment mouse model (correlated with Figure 5A). Mice were orally administered progenitor toxin (L-PTC, 10 ng), and subsequently administered M2+M4 (0.5 μg each) by i.p. injection at 12, 24, or 36 h after the oral administration of L-PTC. *n* = 5 per group. (**B**) Pre-exposure prevention mouse model (correlated with Figure 5B). Mice received i.p. injection of M2+M4 (0.5 μg each) and were then challenged 1, 3, 5, or 7 days later with i.p. administration of 10 i.p. LD_{50} BoNT/B1. Cnt, *n* = 7 per group, M2+M4 (1 day), *n* = 5 per group, M2+M4 (3 days), *n* = 5 per group, M2+M4 (5 days), *n* = 5 per group. M2+M4 (7 days), *n* = 5 per group. Scores were recorded for 2 weeks with symptoms ranging from “no symptoms” to scores of “mild”, “moderate”, and “severe”, defined by an increasing extent of botulism symptoms (fuzzy hair, muscle weakness, limb weakness, and respiratory failure).
